# [Expression of Concern] Roles and mechanisms of action of HNF-4α in the hepatic differentiation of WB-F344 cells

**DOI:** 10.3892/ijmm.2026.5922

**Published:** 2026-07-08

**Authors:** Yumeng Shi, Dehua Zhou, Bingyi Wang, Deren Zhou, Baomin Shi

Int J Mol Med 43: 1021-1032, 2019; DOI: 10.3892/ijmm.2018.4010

Following the publication of this paper, it was drawn to the Editor's attention by a concerned reader that, regarding the images showing periodic acid-Schiff staining in Fig. 5 on p. 1025, the 'PLKO-SH' panel in Fig. 5A contained an overlapping section with the 'WB-F344' panel in Fig. 5C, in spite of the experimental conditions reported for these figure parts being different.

The authors have been contacted by the Editorial Office to offer an explanation for the apparent re-use of the same data in Fig. 5 of this paper, and we are awaiting their response. Owing to the fact that the Editorial Office has been made aware of potential issues surrounding the scientific integrity of this paper, we are issuing an Expression of Concern to notify readers of this potential problem while the Editorial Office continues to investigate this matter further.

